# Impact of neuromyelitis optica spectrum disorder on employment and income in the United States

**DOI:** 10.1002/acn3.52021

**Published:** 2024-02-20

**Authors:** Isabella Gomez Hjerthen, Cristina Trápaga Hacker, William Meador, Ahmed Z. Obeidat, Lucas Horta, Farrah J. Mateen

**Affiliations:** ^1^ Department of Neurology Massachusetts General Hospital Boston Massachusetts USA; ^2^ School of Engineering and Applied Sciences Harvard College Cambridge Massachusetts USA; ^3^ Department of Neurology University of Alabama at Birmingham Birmingham Alabama USA; ^4^ Department of Neurology Medical College of Wisconsin Milwaukee Wisconsin USA; ^5^ Harvard Medical School Boston Massachusetts USA

## Abstract

**Background and Objectives:**

We aim to characterize the sociodemographic and clinical factors associated with loss of jobs, income, and work hours in people with neuromyelitis optica spectrum disorder (NMOSD) in the United States.

**Methods:**

A REDCap‐based survey was administered to working‐age NMOSD patients (18–70 years old) querying demographic information, symptoms, immunosuppression, work hours, income, and caregiver work (11/2022–07/2023). Regression models were developed using MATLAB.

**Results:**

Of 127 participants (97 female; 55% AQP4‐antibody, 19% MOG antibody; 69% Caucasian, 7% Hispanic), with an average diagnosis age of 38.7 years, average disease duration of 6.4 years, mean 3.1 attacks, and 94% of whom were treated with immune system‐directed therapy (53% rituximab, 8% satralizumab, 7% eculizumab, 6% mycophenolate mofetil, 4% inebilizumab, 2% azathioprine, 10% IVIg, 10% other), 56% lost a job due to NMOSD. Employment decreased 12% (80% pre‐ to 68% post‐diagnosis). Thirty‐six percent of participants said they no longer worked outside the home. Significant predictors for post‐NMOSD diagnosis employment status included younger age, lower pain level, no walking aids, and having a job prediagnosis.

Sixty‐eight percent of those employed prediagnosis reduced their work hours, dropping an average of 18.4 h per month since being diagnosed (±10.1 h). Average annual income grew slowly at $1998 during the average 6.4 years of disease duration (14% of the value predicted by the U.S. Bureau of Labor Statistics). Sixty percent of participants had a regular unpaid caregiver; 34% of caregivers changed their work hours or job to help manage NMOSD.

**Discussion:**

We provide a structured analysis of the impact of NMOSD on employment, work hours, and income in the United States, demonstrating its major effect on the livelihoods of patients and their caregivers.

## Introduction

Neuromyelitis optica spectrum disorder (NMOSD) is a rare and potentially disabling autoimmune disease that affects the central nervous system. It is characterized by unpredictable, recurrent episodes of optic neuritis and transverse myelitis, which can lead to cumulative and permanent disability. Estimates indicate that more than 15 000 people in the USA have NMOSD.^1^, ^2^

Several features of NMOSD make it likely to impact employment. NMOSD predominantly affects women and, compared to multiple sclerosis, disproportionately affects people of African American, Asian, and Hispanic backgrounds.^1^, ^2^, ^3^, ^4^ These groups are traditionally not as fully employed or highly paid in the USA.^5^ NMOSD has the cardinal features of visual loss and spinal cord deficits, both of which could be detrimental to work performance and overall employment.^6^ Fatigue, pain, and mental health effects could also lead people with NMOSD to reduce their work hours and time spent outside the home. Frequent medical appointments, hospitalizations, and recurring dosing for immunosuppressive therapy—which also increases NMOSD patients' risk of infection—may further compromise employment stability. While any of these factors could affect people living with NMOSD, little has been investigated on the socioeconomic impact of NMOSD in a large group of patients in the USA. Moreover, the effects on personal income are not well quantified.

Also instrumental to the care of some NOMSD patients is a regular unpaid caregiver. Due to the severity and persistence of symptoms from NMOSD, it is likely that caregiver contributions are high for many people with NMOSD. The extent to which NMOSD affects the employment circumstances of a close relative or friend who functions as a main caregiver is not well reported in the USA either.

Given the limited quantitative analysis of the socioeconomic impact of NMOSD in the USA to date, we designed and implemented a new survey to characterize the impact of NMOSD on employment, work attendance, and wages for people living with NMOSD and their caregivers in the USA. In this study, the NMOSD patient's vantage point provides the perspective for our analyses.

## Materials and Methods

### Ethics approvals

The study protocol was reviewed and exempted as minimal risk by the Massachusetts General Brigham (MGB) institutional review board.

### Survey development

Qualitative work using three focus groups of 20 patients with NMOSD, previously published,^6^ led to the design of the survey used. The instrument included questions on demographic characteristics (age, sex, marital status), household (size, number of adults), pre‐ and post‐diagnosis employment (status, work hours, income), and NMOSD symptoms, treatment, and disease history. The survey was translated into Spanish and Portuguese.

### Participant recruitment and enrollment

Participant recruitment consisted of posts on Rally (an online website for research participation at Massachusetts General & Brigham and Women's Hospitals), letters to people with NMOSD seen at MGB, and advertisements through Twitter, social media from the Sumaira Foundation, and clinical collaborators in Massachusetts, Wisconsin and Alabama.

Participants completed the approximately 30‐minute survey online via RedCap™, either independently or accompanied by a caregiver. The option to fill out the survey by phone with a research staff member was also offered to ensure accessibility for visually impaired individuals and participants without internet access.

Eligibility criteria were diagnosis of NMOSD by a neurologist, current age 18 to 70 years old, and residence in the USA. All participants were asked for proof of NMOSD diagnosis if they were seen outside of the investigators' clinical setting, which was usually provided in the form of a clinical note. The online survey was open for completion from 21 November 2022 to 21 July 2023. Each participant received 75 USD for survey completion.

### Operationalization of variables

Participants were defined as having aquaporin‐4 (AQP4) or myelin oligodendrocyte glycoprotein (MOG) antibody status based on a laboratory test result or a clinical note that documented the positive autoantibody.

Using the U.S. Department of Labor guidelines,^5^ *employment* was defined as working for wages under the permission and regulation of an employer. *Employed* is defined as being paid full‐time (≥40 work hours per week), part‐time, or self‐employed in this study.

For this study's purposes, *income* is money or value received by individuals in exchange for providing goods or services.^7^ All income was queried and reported in United States Dollars.

A *caregiver* was defined as someone who regularly helped the patient take care of the symptoms or disability of NMOSD.

*Fatigue level* and *pain level* were each reported by the participant using an ordinal, categorial scale of zero to four and one to ten, respectively, with four and ten representing more severe symptoms. The level was queried for their state at the time of survey completion.

### Data analysis

The economic vantage point is from the patient's perspective. All demographic, employment, NMOSD history, and symptom data were self‐reported.

Summary statistics were used to depict the demographic and clinical variables of interest, stratified by full‐time employmed, part‐time employmed, and unemployed status groups. Outcomes of interest were post‐NMOSD (1) employment, (2) hours lost, and (3) income lost, at the time of survey completion as compared to before the diagnosis of NMOSD.

### Model development

A binomial logistic regression model was developed to analyze the association of participant‐reported symptoms and employment characteristics with “employed” status. Linear regression models were also used to assess the variables' relationships with postdiagnosis changes in work hours and income.

The predictors evaluated can be found in Appendix A. Those that showed a univariate association with an outcome with *p ≤ 0.2* were included in the first iteration of each model (Appendix A).^8^ The models were then refined using stepwise backward selection, eliminating predictors with *p* > *0.2*. The relevance of two‐way interactions was also analyzed using univariate association. It was determined *a priori* that being female could have a large impact on the regression results; thus, two‐way interactions between gender and (i) race, (ii) years of education, (iii) frequency of NMOSD attacks, (iv) fatigue, and (v) pain level were evaluated. None of these interaction terms were statistically significant predictors in the final model.

The survey form required participants to complete all fields with demographic and key employment and symptom information. This allowed us to use a complete dataset for the main analyses and regressions. Questions about decreased income and work hours for participants that were left blank were counted as zero.

Age was treated as a potential confounder due to an increased likelihood of retirement and unemployment in older populations. Thus, a second version of all models was developed that only included participants under 60 years old and nonretirees (Appendix B). Sensitivity analyses were also conducted to assess the model's validity for full‐time employment alone (vs. our composite definition of employment, which also included part‐time and self‐employment, as provided above).

All statistical analyses were performed using MATLAB version R2022b.^9^ STROBE guidelines were followed.^10^

## Results

There were 130 survey responses. Three participants submitted duplicate surveys, yielding 127 unique responses. Data were reviewed for internal consistency and out‐of‐range responses by the investigators.

### Cohort demographics

Of 127 participants, 97 (76%) were female. Sixty‐nine percent identified as white or Caucasian, 24% as Black or African American, and 7% were Hispanic or Latino. This cohort was mostly English‐speaking; one participant requested the Spanish version of the survey. Almost all participants had received a High School diploma (99%), and 76% had pursued higher education (trade school, college, or professional degree).

### NMOSD characteristics

On average, participants were diagnosed with NMOSD at 38.7 years old (±8.6), had lived with the disorder for 6.4 years (±3.3 (standard deviation)), and had experienced 3.1 attacks (±3.2). Ninety‐four percent reported taking immune‐targeted therapy, the most common of which were B‐cell‐depleting therapies. Table 1 describes the participants' demographic and clinical characteristics.

**Table 1:** Demographic and clinical characterization of study cohort by employment status at the time of participation.

	Total (127)		Employment status					
			Employed full time (62)		Employed part‐time (22)		Unemployed (43)	
Demographic								
Age								
Mean*	45.3	±12.9	43.9	±11.5	35.1	±9.9	52.4	±12.2
Sex								
Female	97	76%	44	71%	17	77%	36	84%
Male	30	24%	18	29%	5	23%	7	16%
Race^a^								
White	88	69%	44	71%	12	55%	32	74%
Black or African American	31	24%	13	21%	8	36%	10	23%
Asian	7	6%	4	6%	3	14%	–	–
Other	5	4%	3	5%	1	5%	1	2%
Ethnicity								
Hispanic or Latino	10	8%	5	8%	2	9%	3	7%
Education^b^								
High School/GED^c^	29	23%	11	18%	5	23%	13	30%
Trade School	7	6%	4	6%	1	5%	2	5%
College/Bachelor's Degree	54	43%	28	45%	11	50%	15	35%
Professional Degree	35	28%	19	31%	5	23%	11	26%
Marital status								
Single	32	25%	15	24%	8	36%	9	21%
Married/Partnership	82	65%	45	73%	11	50%	26	60%
Divorced/Separated	13	10%	2	3%	3	14%	8	19%
Household								
Mean size*	3.1	±1.6	3.2	±1.3	3.5	±1.6	2.7	±1.1
Members earning income*	1.7	±0.9	1.7	±0.8	2.0	±0.9	1.3	±0.9
Clinical								
General information								
Years since diagnosis*	6.5	±4.8	5.8	±4.1	5.4	±4.6	7.9	±5.6
History of myelitis	73	58%	33	53%	12	55%	28	65%
History of optic neuritis	47	37%	21	34%	8	36%	18	42%
Use walking aid(s)	29	23%	9	15%	2	9%	18	42%
Fatigue due to NMOSD	107	84%	47	76%	21	95%	39	91%
Pain due to NMOSD	94	74%	38	61%	17	77%	39	91%
Antibody status^d^								
AQP4	71	56%	35	56%	9	41%	27	63%
MOG	25	20%	13	21%	4	18%	8	19%
Double seronegative	30	24%	13	21%	9	41%	8	19%
Treatments^e^								
Azathioprine	2	2%	–	–	1	5%	1	2%
Eculizumab	9	7%	4	6%	2	9%	3	7%
Inebilizumab	5	4%	4	6%	–	–	1	2%
IVIg	12	10%	6	10%	2	9%	4	9%
Mycophenolate mofetil	7	6%	3	5%	1	5%	3	7%
Rituximab	67	53%	36	58%	8	36%	23	56%
Satralizumab	10	8%	3	5%	4	18%	3	7%
Other^f^	13	10%	3	5%	4	18%	6	14%
None	7	6%	5	8%	–	–	2	5%
Comorbidities								
Asthma	15	12%	8	13%	3	14%	4	9%
Diabetes	4	3%	2	3%	1	5%	1	2%
Gastrointestinal disease	12	9%	3	5%	–	–	9	21%
Hypertension	16	13%	8	13%	2	9%	6	14%
Lung disease	6	5%	1	2%	2	9%	3	7%
Obesity	25	20%	13	21%	5	23%	7	16%
Sjögren Syndrome	10	8%	5	8%	–	–	5	12%
Systemic lupus erythematosus	5	4%	–	–	–	–	5	12%
Other^g^	49	39%	19	31%	4	18%	26	60%
None	49	39%	25	40%	9	41%	15	35%

* ± indicates standard deviation.

^a^ Four participants selected more than one race.

^b^ Two participants completed up to Secondary School (both AQP4+ antibody status).

^c^ General Education Diploma (GED).

^d^ One participant had unknown antibody status.

^e^ Five participants were on multiple therapies.

^f^ IGSC (*n* = 1), prednisone (*n* = 4), unknown infusion (*n* = 3).

^g^ Includes hypothyroidism (*n* = 5), cardiovascular disease (*n* = 1), arthritis (*n* = 1).

### NMO symptoms and impact on work

Over half of the study participants (57%) had a history of myelitis, and 37% had optic neuritis. Most reported feeling at least some fatigue due to NMOSD (85%), and 50% said they were fatigued “often” or “almost always.” Fifty‐one percent of participants stated that fatigue negatively affected their performance at work. Seventy‐four percent of participants experienced pain due to NMOSD; of this group, 69% said it affected their ability to work, and 57% said it influenced their decision to work.

For 56% of participants, NMOSD interfered with their daily activities moderately to severely, and 9% said it completely prevented them from completing everyday tasks (mean = 4.5 ± 3.2; scale of 0–10 [0 = least hindrance, 10 = complete hindrance]).

Mental health impact was noted: 24% reported that feeling little interest or pleasure in doing things affected their ability to do work about half of the days since their diagnosis, and 11% faced this challenge every day. Thirty‐five percent of participants felt that being down, depressed, or hopeless on half or more of the days since their NMOSD diagnosis had affected their completion of job duties. Pre‐ and post‐NMOSD diagnosis employment status, weekly work hours, and annual income are provided in Table 2.

**Table 2:** Overview of pre‐ and post‐NMOSD diagnosis employment of study participants.

	Pre‐NMOSD		Post‐NMOSD	
Employed				
Yes	101	80%	83	65%
No	26	20%	44	35%
Employment status				
Paid full time (40 hr/wk)	64	65%	55	43%
Student	12	9%	2	2%
Self‐employed	11	9%	7	6%
Paid part‐time	11	9%	22	17%
Retired	3	2%	12	10%
Disability status	3	2%	22	17%
Unemployed, looking for work	2	2%	5	4%
Unemployed, not looking for work	2	2%	2	2%
Homemaker	2	2%	–	–
Employment				
Weekly work hours	33.9	±17.9	15.8	±17.4
Annual income	53, 888	±49, 902	55, 887	±57, 041

Overall, 56% of study participants reported losing a job since being diagnosed with NMOSD. Employment decreased 12% (from 80% prediagnosis to 68% postdiagnosis), suggesting that even though some participants gained a new job after the initial loss, a smaller proportion of this cohort was employed postdiagnosis. Additionally, 68% of those who were employed both pre‐and post‐diagnosis reduced their work hours, losing an average of 18.4 hours per month (±10.1) due to symptoms or other NMOSD‐related factors. Thirty‐six percent of participants said they no longer worked outside the home. A minority, 19 participants (15%), reported having disability status.

### Impact on income

This cohort's annual income remained almost constant from the time of diagnosis to the time of survey completion, but there was wide variability, increasing $1998.70 USD on average (±51 465.03) overall. Thirty‐eight percent of participants reported losing at least some of their income (mean = $40 499.32 ± 35 525.83), and 10% said they had completely lost earnings. The 51% of participants who saw an increase in income between the time of their NMOSD diagnosis and time of study participation (mean = 6.4 years ± 3.3) reported earning around $33 630 more on average (±43 758). Eighty‐four percent of participants who gained income were female, a slightly greater percentage than that of the study cohort. Eighty‐four percent identified as white or Caucasian and 18% as black or African American (the percentage exceeds 100 because one participant identified as both). On average, these participants had experienced 2.5 NMOSD attacks (±2.2), almost one less than the cohort's average.

### Unpaid caregivers

Sixty percent of participants had a regular unpaid caregiver, 96% of whom were part of the nuclear family (e.g., spouse, child, sibling, or parent). Thirty‐four percent of caregivers changed their work hours or job to help the participant manage NMOSD. On average, caregivers who decreased their amount of work lost 12.4 h per month (±14.2), while those who increased their hours added 26.3 h per month (±17.0). Table 3 details the distribution of caregiver employment change post‐NMOSD diagnosis.

**Table 3:** Caregiver demographics and employment change due to NMOSD‐related responsibilities.

	Total (127)		Employment status					
			Employed full time (62)		Employed part‐time (22)		Unemployed (43)	
Caregiver								
Yes	78	61%	37	60%	11	50%	30	70%
Spouse/partner	50	66%	28	45%	6	55%	16	53%
Parent(s)	19	25%	7	19%	3	27%	9	30%
Child(ren)	5	7%	2	5%	1	9%	2	7%
Friend(s)	3	4%	–	–	–	–	2	7%
Sibling(s)	1	1%	–	–	–	–	1	3%
No	49	39%	25	40%	11	50%	13	30%
Caregiver employment change^a^								
Temporary stop	12	15%	6	16%	2	18%	4	13%
Switched jobs	7	9%	1	3%	2	18%	4	13%
Reduced hours	6	8%	1	3%	2	18%	3	10%
Mean decrease*	10.9	±12.9	17.6	–	12.5	±5	22	±16.4
Increased hours	2	3%	–	–	1	9%	–	–
Mean increase*	10.5	±16.7	–	–	30	–	–	–
No change	53	68%	29	78%	6	55%	18	60%

^a^ 1 participant's caregiver reduced and increased their work hours since diagnosis.

* ± indicates standard deviation.

### Analyses for association

Table 4 shows the adjusted odds ratios (aORs) for predictors of employment, work hours lost, and income lost in the study cohort. In the case of hours and income, a positive outcome indicated that the participant lost income or work hours, while a negative outcome indicated gain.

**Table 4:** Logistic regression model predicting “employed” status, and linear regression models predicting work hours and income lost by participants with NMOSD.

		Outcome	Predictors	Regression analysis
		Odds ratio	*p*‐value	95% CI
Currently employed^a^	Age	0.89	<0.001**	0.84–0.94
	Pain	0.78	<0.03*	0.62–0.97
	Disease duration	0.90	0.07	0.80–1.01
	Employed at diagnosis	6.92	0.007**	1.71–28.03
	Walking aids	0.30	0.04*	0.09–0.97
	Annual cost of NMOSD	1.0001	0.19	1.0000–1.0002
	Feeling depressed	0.43	0.003**	0.24–0.75
Work hours lost^b^	Age	1.69	<0.001**	1.25–2.29
	Pain	6.92	0.03*	1.24–38.63
	Walking aids	8.4 × 10^4^	0.02*	4.92–1.4 × 10^9^
	Effect on productivity	3.18	0.07	0.93–10.93
		***β***	***p*‐value**	**95% CI**
Income lost^c^	Age	900	0.005**	−29, 800–31, 600
	Prediagnosis income	0.35	<0.001**	0.20–0.51
	Effect on daily tasks	5, 602	<0.001**	2, 747–8, 457
	Feeling depressed	9, 352	0.035*	758–17, 946

^a^ Adj. *R* ^2^ = 0.48.

^b^ Adj. *R* ^2^ = 0.26.

^c^ Adj. *R* ^2^ = 0.36.

*p< 0.05, **p < 0.01.

Younger age, less pain, and being employed at the time of diagnosis were associated with a higher chance of employment post‐NMOSD diagnosis. Older age, the use of walking aids, and a greater self‐perceived impact of NMOSD on productivity led to more work hours lost per month. Finally, older age, lower prediagnosis income, the interference of NMOSD with daily tasks, and frequent feelings of depression were associated with increased income loss.

### Sensitivity analyses

When constraining the logistic regression outcome for full‐time employment only, age and “employed” status at diagnosis still decreased the participant's probability of employment (*p < 0.01*). A self‐perceived increased effect of NMOSD on daily activities also reduced the probability of current employment (*p < 0.05*).

Including only participants under 60 years old showed that age, higher pain level, not having “employed” status at diagnosis, and use of walking aids were still statistically significant predictors of unemployment (Appendix B). When currently retired participants of any age were excluded, only increased pain level was no longer significant (Appendix B).

When excluding MOG antibody‐positive participants, a higher frequency of feeling “little to no interest in doing things” was a significant predictor of unemployment (*p < 0.05*). Unlike in the complete cohort, higher pain level was not a significant predictor of unemployment in for AQP4 antibody‐positive and double seronegative participants.

Constraining the income model to participants younger than 60 years old showed that pre‐NMOSD diagnosis income and perceived NMOSD effect on productivity continued to be significant predictors of lost income. For AQP4‐antibody positive or double seronegative participants, income pre‐NMOSD diagnosis and interference of NMOSD with daily activities were the two significant predictors of income loss (Appendix B).

In participants under 60 years old, age and the use of walking aids were still statistically significant predictors of a greater amount of work hours lost. An increased perceived effect of NMOSD on productivity at work also significantly decreased work hours for participants who had not retired. Among AQP4‐antibody positive or double seronegative study participants, age was the only predictor of lost work hours with *p < 0.05*.

## Discussion

This survey‐based study aimed to assess the socioeconomic impact of NMOSD as self‐reported by people living with the disease in the USA. We measured three relevant endpoints in this assessment, finding that NMOSD significantly affects each of employment, wages, and work hours. This assessment found that 32% of participants are unemployed and 56% have lost a job due to NMOSD. By comparison, the U.S. unemployment rate as of June 2023 was 3.6%, a 20‐year low.^11^ Despite the positive fact that most participants were employed at the time of the study, NMOSD was found to have myriad effects on employment: 36% of participants had stopped working outside the home, the number of part‐time employed participants doubled postdiagnosis, and 18.4 monthly work hours (±10.1) were lost by working participants on average.

Both clinical and demographic variables were significant predictors of employment postdiagnosis, and included prediagnosis employment, younger age at diagnosis, lower pain scores, and not needing walking aids. Predictors of wages and work hours lost were similar to those for employment. Given the many ways NMOSD can affect an individual, it is notable that myelopathic symptoms predominanted clinically, including need for walking aids and pain. Whereas NMOSD is not generally categorized as a disorder of pain, it is relevant here that pain has an important impact on socioeconomic functioning. It is not surprising that prediagnosis employment was associated with postdiagnosis employment. Given the overall well‐educated group of participants in this study, it is possible that higher education is protective for future employment, but we were unable to identify this in our cohort. Since younger age is also associated with fewer disease attacks in most participants, the accrual of disease attacks over time may also worsen employment status. This is consistent with qualitative work done on NMOSD and employment that suggests that patients with visual loss may find accommodations but *both* visual loss and spinal cord dysfunction make them unable to find any work.^6^ Conversely, younger patients may be more willing to learn new skills, pivot in their job tasks, be more computer‐based, have better access to immunosuppression over their disease course, and/or have less income to lose.

Our study is consistent with others who have identified a high unemployment rate and limited full‐time employment among study participants with NMOSD. Prior work on NMOSD found 11% to 35% of participants from the United States, Spain, and United Kingdom were employed.^12^, ^13^, ^14^ However, these prior studies did not specifically aim to characterize employment of NMOSD patients and had study populations that were about 6 years older on average. This inevitably increased the number of retired participants included. Furthermore, the impact of the COVID‐19 pandemic—which forced work from home, caused layoffs of some workers, and increased the risk to people with several forms of immunosuppression—is notable in this survey. The currently tight labor market and low U.S. unemployment rate may be increasing Americans' job prospects, including people with NMOSD. At this time, it is commonly recognized that most people who want a job in the USA could find one.^5^ The transition to remote work could also be making jobs more accessible, allowing people with decreased mobility, vision loss, and other symptoms related to NMOSD to work from home. Since the average time living with NMOSD was around 6 years (mean = 6.4 years ± 3.3), these external influences over the years of the COVID‐19 pandemic are relevant. Whether employment is at its optimal level for NMOSD alongside the U.S. population will require longitudinal studies of NMOSD and employment.

We also found that on average, participants earned $53 888 (±$49 902) before being diagnosed. The U.S. Bureau of Labor Statistics Consumer Price Index (CPI) Inflation Calculator^15^ predicts a gain of $13 994 (25.97%) during these 6.4 years; however, this cohort had an average increase of $1998, which is 14% of the predicted value. Decreased full‐time employment, frequent job changes, and constant difficulties completing daily tasks affected this cohort's ability to dedicate time to work and earn income. In addition to age, prediagnosis income, and a higher impact of NMOSD on daily tasks, feeling down, depressed, or hopeless was found to increase the income lost by participants in this cohort. This suggests that both NMOSD symptoms and their effects on mental health hindered people with the disease in the workforce. Since NMOSD is not considered to be a disease of mood or concentration, there are few clinical assessments or support programs specifically available to address mood in NMOSD. Since feeling down, depressed, and hopeless and difficulty completing tasks may not be directly related to the disease process, our results are relevant to efforts that could improve engagement and reintegration of people with NMOSD into the workforce in some cases. Programs to reduce job loss and provide resilience training are being actively studied in related diseases, including multiple sclerosis.^16^

Employment instability due to NMOSD extended beyond the diagnosed person, since 60% of study participants reported having a regular unpaid caregiver, more than a third of whom changed their work hours or job to help the patient manage NMOSD. Similar to a previous study conducted in the United Kingdom,^13^ the majority of caregivers were part of the person with NMOSD's nuclear family (94%). Importantly, the variations in caregivers' behaviors are high. While some stopped working to provide care and to support the person with NMOSD, others increased work hours, presumably to focus on income for the household and covering increased expenses. The needs of the household were clearly not uniform among participants with NMOSD. In 2022, Hughes *et al*. quantified the health utilities and costs for NMOSD, finding that the main contributor to direct nonmedical cost incurred was informal care (19 432 USD per year).^13^ On average, caregivers in their study worked 0.3–4.4 h less per week. Since it is common to have a caregiver in NMOSD, this additional cost is a significant consideration that should be investigated further. We did not specifically ask about lost income of the caregiver in NMOSD in this study given the extended length of the survey and the survey respondent being the patient herself; however, income lost or increased due to NMOSD caregiving may provide yet another angle by which to view the complex socioeconomic impact of NMOSD beyond the patient.

We stratified analyses of employment, hours lost, and income lost in the disease subgroups of AQP4‐positive, MOG‐positive, and double seronegative participants. It was difficult to detect important differences in these subgroups, likely because our sample size was small when separated into three groups. We also studied the subgroup of *full* employment, and our results remained stable. Similarly, the results remained essentially unchanged by confining our study to those between 18 and 60 years old, that is, those who would not be of usual retirement age.

We are aware of one other study of NMOSD patients in the USA that included a survey of employment. A 2019 report^12^ showed that 67 of 193 NMOSD participants (35%) in the CIRCLES study were currently employed, including both full‐ and part‐time work. Of those who were unemployed in that study, 64% reported disability status, and 5% were looking for work. On average, participants of the CIRCLES study with NMOSD were 49.2 years old (±12.8)—more than 4 years older than this study's cohort—which could have resulted in a greater number of retired and more severely disabled participants.

It is unclear how well our results in a Western high‐income population generalized to countries elsewhere. In Western China, Han *et al.* recently studied 351 females with NMOSD, finding lower education (middle school or below), older age, higher annual disease recurrence rate, and severe disability were each predictors of unemployment.^17^ Similar to our study, 54% of participants reported that NMOSD led to job loss. Education level was not a significant predictor of unemployment in our study; however, our cohort had less variation, since all except one participant in our cohort had completed high school, while 59% of participants in Han *et al*.'s study completed high school or above. This may also be a factor that influenced the relatively low unemployment rate in our cohort: since the data were collected from a generally more educated population, participants are at less risk of losing a job than people who have a lower education level and have high‐replacement roles and often, more physically demanding tasks.

Our study had several limitations, including convenience sampling of people with NMOSD. The study was not population‐based, making the generalizability of our findings to the entire NMOSD patient population in the USA uncertain. While we studied a wide range of factors, we did not test the number of jobs in participants' lifetime and the current duration of participants' jobs. We did not ask for specific occupations. We included MOG antibody disease in our study, although it is becoming recognized as a distinct disease from aquaporin‐4 seropositive and double seronegative disease. Important differences in the age of onset of NMOSD (e.g., in the pediatric age range) may not have been captured in our sample, and these could also affect employment. There is also a potential bias in responses, since the ability and willingness to complete a survey online regarding employment is required. The study allowed for telephone‐based responses for participants with low vision or no computer, but these situations may still have been prohibitive factors for reading recruitment letters or verifying the NMOSD diagnosis if people were not seen within our hospital practice. Similarly, there may have been a response bias, such that employed people wanted to fill out a survey about employment more than those who were unemployed. We attempted to mitigate this limitation by paying each participant 75 USD for her or his time, incentivizing the survey response across employment situations. We do not measure the relative contribution of specific NMOSD disease factors to unemployment as reported by each individual (e.g., fatigue 50%, pain 30%, and mobility 20%), noting this would be useful for future programming to assist people with NMOSD.

Strengths of our work include the enrollment of participants across many U.S. states, recruited from diverse settings and situations. Our sample size is relatively high for a rare neurological disease. We provide an in‐depth study of multiple issues in employment, for which participants took approximately 30 min filling out the survey to reach this depth and saturation of information. Each participant had diagnostic verification, and given the online nature of the study forms, missing data were limited. Data were analyzed to provide information relevant to future NMOSD patients and allow for a detailed assessment of potential demographic, household, microeconomic, symptomatic, and disease factors that can each be studied further. Our cohort also provides a current snapshot of the disease modifying treatment, symptoms, economic status, and caregiver burden of NMOSD in the USA. We underscore known unmet needs in the NMOSD field including the work‐altering burden of pain, fatigue, mental health, and coping with the disease while emphasizing their at times surprising magnitude in surveyed patients.

To our knowledge, patient‐reported microeconomic studies dedicated to work, wages, and caregiver economics in United States NMOSD patients are few in number. While most people with NMOSD in this study were employed, there were important aspects of working from home, lower work hours, and static income in many participants. There are varying effects of a severe neurological disease on caregivers, even in patients who are optimally treated with a DMT. Illustrating the current living conditions for people living with NMOSD in the USA could shape future strategies for employers, policymakers, and patient‐focused foundations, aiming to improve their overall well‐being.

## Disclaimers

Dr. Mateen has received consulting fees from Alexion, EMD Serono, Genentech, Horizon Therapeutics, and TG Therapeutics. She has received research grants from Biogen, Genentech, Horizon Therapeutics, and Novartis. Dr. Obeidat received speaking or consulting fees from Alexion, Banner Life Sciences, Biogen, Biologix, Bristol Myers Squibb, Celgene, EMD Serono, Genentech, GW Pharma, Horizon Therapeutics, Jazz Pharma, Novartis, Sanofi/Genzyme, Sandoz Pharmaceuticals, TG therapeutics, VielaBio, and Honoraria from Medscape and MJH Life Sciences.

## Funding Information

Investigator‐Initiated Grant from Horizon Therapeutics.

## Conflict of Interest

IGH: data collection, analysis, interpretation; writing; editing for critical intellectual content. CTH: data collection. WM: data collection. AZO: data collection. LH: data collection. FJM: data collection, interpretation; writing, editing for critical intellectual content, study supervision, obtaining funding.

## Supporting information


Appendix A.

Appendix B.

## Data Availability

Qualified research personnel may obtain de‐identified data collected during this study in accordance with institutional review board policies, upon request.
