# Supplementary material for: Impact of neuromyelitis optica spectrum disorder on employment and income in the United States
Source: Ann Clin Transl Neurol. 2024 Feb 20;11(4):1011–20. doi: 10.1002/acn3.52021 (PMC11021617; doi:10.1002/acn3.52021)
Supplement: Supplementary file 1 — Appendix A. [file ACN3-11-1011-s001.docx]

**Appendix A:** Demographic, Clinical, and Self-Assessment Variables by Respondents with NMOSD for Predictive Model Building for each of (i) Employment, (ii) Lost Work Hours, and (iii) Lost Income, using a threshold of p<0.20 for univariate testing

Predictor Outcome

Currently employed Work hours lost Income lost

|  |  | */3* | p-value | */3* | p-value | */3* | p-value |
| --- | --- | --- | --- | --- | --- | --- | --- |
|  | Age | -0.07 | < 0.001** | 0.61 | < 0.001** | 987 | 0.005** |
|  | Sex | -0.71 | 0.14 | -4.65 | 0.38 | -1, 789 | 0.87 |
|  | Fatigue | -0.57 | 0.002* | 5.62 | 0.002** | 14,146 | < 0.001** |
|  | Years of education | -0.04 | 0.27 | -0.001 | 0.99 | -365 | 0.67 |
|  | Education level | 0.25 | 0.12 | -1.52 | 0.44 | -4, 321 | 0.28 |
|  | Pain | -0.29 | < 0.001** | 3.64 | < 0.001** | 7,734 | < 0.001** |
|  | Attacks | -0.01 | 0.74 | -0.09 | 0.84 | 677 | 0.48 |
|  | MOG-positive | 0.16 | 0.73 | -8.3 | 0.14 | -16, 074 | 0.16 |
|  | AQP4-positive | -0.46 | 0.23 | 7.83 | 0.08 | 16,212 | 0.08 |
|  | Disease duration | -0.09 | 0.02* | -0.14 | 0.77 | -1, 514 | 0.11 |
|  | Time undiagnosed | -0.023 | 0.41 | 0.001 | 0.99 | 4.42 | 0.97 |
|  | Employed at diagnosis | 0.61 | 0.17 | 33.13 | < 0.001** | 49,191 | < 0.001** |
|  | Unpaid care | -0.36 | 0.35 | 3.25 | 0.48 | 12,796 | 0.17 |
|  | Walking aids | -1.49 | < 0.001** | 21.73 | < 0.001** | 29,048 | 0.007** |
|  | NMOSD expenses | 6.4 X 10-5 | 0.12 | 0.0003 | 0.47 | 0.87 | 0.22 |
|  | Days since last attack | 2.5 X 10-5 | 0.56 | -0.0006 | 0.14 | -0.44 | 0.60 |
|  | Effect on productivity | -0.14 | 0.005** | 2.1 | < 0.001** | 5,070 | < 0.001** |
|  | Effect on daily tasks | -0.22 | < 0.001** | 1.89 | 0.007* | 6,624 | < 0.001** |
|  | Low interest | -0.59 | 0.002** | 4.67 | 0.02* | 17,389 | < 0.001** |
|  | Feeling depressed | -0.42 | 0.02* | 3.71 | 0.07 | 16,291 | < 0.001** |
|  | Two-Way Interactions |  |  |  |  |  |  |
|  | Race : Female | 0.17 | 0.89 | 9.86 | 0.50 | -11, 132 | 0.62 |
|  | Years of education : Female | -0.04 | 0.64 | -0.71 | 0.49 | -4, 012 | 0.06 |
|  | Attacks : Female | 0.02 | 0.88 | 1.80 | 0.20 | -1, 174 | 0.69 |
|  | Fatigue : Female | 1.47 | 0.06* | -7.92 | 0.06* | -21, 218 | 0.01** |
|  | Pain : Female | 0.09 | 0.68 | 0.42 | 0.81 | -2, 588 | 0.48 |
