# Supplementary material for: Impact of neuromyelitis optica spectrum disorder on employment and income in the United States
Source: Ann Clin Transl Neurol. 2024 Feb 20;11(4):1011–20. doi: 10.1002/acn3.52021 (PMC11021617; doi:10.1002/acn3.52021)
Supplement: Supplementary file 2 — Appendix B. [file ACN3-11-1011-s002.docx]

Appendix B: Sensitivity Analyses, restricted to the subcohorts (i) <60 years old, (ii) not retired, and (iii) either aquaporin-4 antibody seropositive or double seronegative

Outcome Predictors Regression Analysis

|  | | Odds ratio | p--value |
| --- | --- | --- | --- |
| Employed full time |  |  |  |
|  | Age | 0.96 | < 0.02* |
|  | Employed at diagnosis | 5.94 | 0.003** |
|  | Effect on productivity | 083 | 0.02* |
|  | Effect on daily tasks | 0.81 | 0.02* |
| Currently employed |  |  |  |
|  | Age | 0.90 | < 0.002** |
|  | Pain | 0.78 | < 0.06 |
| < 60 years old | Employed at diagnosis | 8.71 | 0.009** |
|  | Walking aids | 0.18 | 0.006** |
|  | Effect on productivity | 0.85 | 0.08 |
|  | Age | 0.90 | < 0.001** |
|  | Employed at diagnosis | 11.07 | 0.002** |

Not retired

Walking aids 0.24 0.02* Annual cost of NMOSD 1.0001 0.19

Effect on productivity 0.79 0.01* Effect on daily tasks 0.77 0.03* Age 0.91 < 0.001••

Employed at diagnosis 7.28 0.007**

| AQP4-positive  and double | Walking aids | 0.24 | 0.02* |
| --- | --- | --- | --- |
| seronegative | Effect on productivity | 0.79 | 0.01* |
|  | Disease duration | 0.88 | 0.05* |
| Work hours lost | Lack of interest | 0.46 | 0.01* |
|  | Age | 2.14 | < 0.001** |
| < 60 years old | Pain | 4.85 | 0.13 |
|  | Walking aids | 6.6 X 10^4^ | 0.05* |
|  | Effect on productivity | 2.93 | 0.17 |
|  | Age | 1.85 | < 0.001** |
| Not retired | Pain | 4.30 | 0.12 |
|  | Walking aids | 1.9 X 10^5^ | 0.02* |
|  | Effect on productivity | 4.87 | 0.03* |
|  | Age | 1.41 | < 0.05* |
| AQP4-positive  and double | Pain | 5.20 | 0.08 |
| seronegative | Walking aids | 1.1 X 10^4^ | 0 08 |
|  | Effect on productivity | 3.76 | 0.06 |
| Income lost |  | */3* | p--value |
|  | Age | 562 | 0.20 |
| < 60 years old | Pre-diagnosis income | 0.51 | < 0.001** |
|  | Effect on daily tasks | 7,364 | < 0.001** |
|  | Feeling depressed | 6,033 | 0.21 |
|  | Age | 923 | < 0.01* |
| Not retired | Pre-diagnosis income | 0.36 | < 0.001·• |
|  | Effect on daily tasks | 6,618 | < 0.001** |
|  | Feeling depressed | 8,896 | 0.05* |
|  | Age | 479 | < 0.12 |
| AQP4-positive  and double | Pre-diagnosis income | 0.21 | < 0.004** |
| seronegative | Effect on daily tasks | 3,758 | < 0.007** |
|  | Feeling depressed | 9,498 | 0.03* |
